# Network Analysis–Driven Machine Learning Model for Identifying High-Cost Stroke Inpatients Using Hospital Discharge Data: Retrospective Study

**DOI:** 10.2196/93680

**Published:** 2026-08-10

**Authors:** Haohui Shen, Yilong Yang, Mengge Zhang, Jingyi Xiang, Runan Wang, Pin Yao

**Affiliations:** 1 Department of Health Policy and Management, Hangzhou Normal University, Hangzhou, Zhejiang, China; 2 Department of Pediatrics, Shengjing Hospital of China Medical University, Liaoning, Shenyang, China; 3 Department of Health Management, Shenyang Women's and Children's Hospital, No.87 Danan Road, Shenyang, Liaoning, 110011, China, +86-18940056788, +86-024-22853728

**Keywords:** stroke, high-cost patients, machine learning, comorbidity network, network analysis, hospital discharge data

## Abstract

**Background:**

The medical burden caused by stroke is increasingly severe, and a small minority of high-cost patients consume the majority of medical expenditures. Therefore, revealing the formation mechanisms of this population and exploring a scientific cost-risk stratification system are crucial for improving the quality of care and achieving the optimal allocation of medical resources.

**Objective:**

This study aimed to construct a comorbidity network for patients with stroke using standardized front-page medical record data, extract network features that reflect complex disease interactions, and develop identification models in combination with machine learning algorithms. The study focused on building a core model integrating variables from the near-discharge stage for stratifying the risk of high hospitalization costs in patients at the near-discharge stage. In addition, an early prediction model was developed using only data available at admission.

**Methods:**

We conducted a retrospective study, collecting the hospital discharge data of inpatients with stroke from a tertiary hospital in Northeast China between 2021 and 2023. The data from 2021 to 2022 were used to construct a network and extract features to capture the potential relationship between diseases and high costs. Using the 2023 data partitioned into training and testing sets, we developed 5 models to identify inpatients with stroke who incurred high hospitalization costs and compared their performance when input with different features. In addition, the Shapley Additive Explanations interpretability method was adopted to explain the global and local contributions of the model features.

**Results:**

The inclusion of network features significantly improved the model’s performance, among which Extreme Gradient Boosting performed the best. The global feature importance showed that network features occupied a major proportion. The results of the Shapley Additive Explanations interaction analysis indicated potential phased changes in patient resource consumption. However, the overall performance of the early identification model constructed solely from admission data was subject to clear limitations.

**Conclusions:**

This study developed an integrated framework combining comorbidity network analysis with machine learning, which significantly improved the accuracy of identifying inpatients with stroke at high risk of incurring excessive hospitalization costs. The core model demonstrated good performance in risk stratification during the near-discharge stage, showing potential for application in the formulation of risk management strategies and the optimization of health care resource allocation. It also laid the foundation for the subsequent development of more accurate early identification models.

## Introduction

According to the 2021 Global Burden of Disease study, stroke remains the second leading cause of death worldwide [1]. It is estimated that the number of stroke-related deaths will increase by 50% between 2020 and 2050, with this health burden predominantly borne by low- and middle-income countries [2]. In China, the economic burden of stroke is particularly heavy; in 2018, its direct treatment costs reached $58.6 billion, of which hospitalization expenses accounted for 79.03% [3]. Notably, the consumption of medical resources typically exhibits a significantly skewed distribution, with disproportionate medical expenditures often concentrated within a small group of high-cost patients [4]. This phenomenon of cost concentration is prevalent worldwide [5-7]. Although interventions, such as interdisciplinary transitional care and complex care management, aim to control costs by optimizing services [8-11] and have been proven to have certain short-term effects [12,13], many high-cost patients still face issues of overtreatment or inefficient care [14]. In this context, implementing rational risk stratification according to patients’ overall disease conditions could potentially address existing shortcomings, ultimately leading to improved cost containment and the optimal allocation of health care resources.

Owing to its powerful data processing capacity, machine learning has been extensively used in research concerning health care expenditure prediction. For example, Ma et al [15] predicted the average daily costs of patients with psychiatric disorders, Hu et al [16] identified the determinants of high costs among patients with breast cancer, and Osawa et al [17] developed a predictive model for high-need, high-cost patients. Although previous studies have confirmed the utility of machine learning in predicting medical costs and identifying high-cost patients, it remains necessary to extract latent features closely related to medical expenditures from limited datasets to further enhance model performance. Stroke is typically characterized by a high incidence of multiple comorbidities [18], making the effective use of patients’ rich comprehensive diagnostic information crucial. Complex comorbidities not only increase the difficulty of treatment and the risk of mortality but are also closely associated with significant escalations in hospitalization costs [19,20]. Although existing predictive models generally incorporate comorbidities as key features [21-23] regarding feature processing, most studies rely on rule-based scoring systems (eg, the Charlson comorbidity index [CCI] and the Elixhauser comorbidity index [ECI]) [24,25] or simple disease counts. These traditional methods focus solely on the linear accumulation of comorbidities, ignoring the intricate interactions among diseases, which leaves latent information in the data untapped and thereby limits the models’ ability to identify high-cost patients.

To overcome the aforementioned methodological limitations, network analysis provides a systematic methodological framework. This network-based analytical paradigm goes beyond traditional simple disease counting; by integrating multiple quantitative indicators such as correlation coefficients, odds ratios (ORs), and the Salton cosine index [26], it can effectively evaluate the strength of associations between diseases and subsequently construct comorbidity networks [27,28]. This topological perspective not only helps identify frequently co-occurring disease clusters but also further reveals the potential interactions between diseases. With the widespread application and standardization of the *International Classification of Diseases, Tenth Revision* (*ICD-10*) codes, it has become possible to construct phenotypic comorbidity networks (PCNs) using hospital discharge data. For example, Xu et al [29] improved the prediction accuracy of self-harm behavior by incorporating comorbidity network features, while Hu et al [30] effectively predicted patients’ length of stay (LOS) using a multiplex network and a patient similarity network. Notably, Yang et al [31] constructed a dual network using the diagnostic records of patients with ischemic heart disease, revealing that the extracted network features significantly outperformed traditional comorbidity indices, thereby effectively enhancing the model’s ability to identify high-cost patients.

To the best of our knowledge, network analysis has not yet been applied to the identification and risk characterization of high-cost inpatients with stroke. Given that stroke, as the second leading cause of death globally, imposes a profound resource burden on health care systems, addressing this research gap holds significant practical relevance. Furthermore, previous studies have largely focused on improving overall model performance while often neglecting the interpretability of internal decision-making mechanisms, thereby failing to fully elucidate the specific contributions of individual features to medical costs and their association patterns. Therefore, this study aimed to construct a comorbidity network based on data from the medical record front pages of discharged patients, extract topological features that characterize the complex interactions among diseases, and subsequently build a near-discharge classifier to verify the applicability of network analysis in this research field. Simultaneously, by introducing the Shapley Additive Explanations (SHAP) analysis method, this study is dedicated to developing a robust and clinically interpretable evaluation framework, aiming to provide valuable references for the cost-risk stratification of patients with stroke, thereby offering a scientific basis for hospital administrators to formulate more refined and targeted cost control strategies.

## Methods

### Data Sources and Preprocessing

This retrospective study used discharge data from a tertiary hospital in Northeast China. The data collection period was from January 1, 2021, to December 31, 2023. The dataset contains comprehensive information on inpatients, including demographic characteristics (such as age and gender), diagnostic information at admission, including primary and secondary diagnoses, admission and discharge status, and total hospitalization costs. On the basis of the *ICD-10* coding system, this study screened for patients with stroke and ultimately included inpatient cases with a primary diagnosis code of I60, I61, or I63. To minimize information bias and ensure data quality, this study established a rigorous data screening and preprocessing workflow. During the data quality control phase, extreme cases with a LOS of less than 24 hours or more than 90 days were first excluded. Subsequently, a systematic evaluation of the missingness of key features was conducted. To balance data integrity and sample representativeness, as well as to prevent the introduction of potential systematic bias from overreliance on data imputation, variables demonstrating a missingness rate greater than 20% were eliminated [32]. Among the initially extracted raw features, a total of 2 variables were excluded for exceeding this threshold; the remaining variables exhibited good data completeness, with missing proportions ranging from 0.13% to 1.95%. Statistical evaluation confirmed that the missing data in this study satisfied the assumption of missing at random, and no statistically significant differences in missingness rates were detected across the various cost groups. To impute the remaining missing values, this research used the K-Nearest Neighbors (KNN) imputation technique, a method that estimates missing data by leveraging local structural similarities across samples, thereby effectively retaining the original data’s distributional properties and the underlying associations among variables. To avoid data leakage from distance-based imputation, we fitted the KNN imputer using only the 2021 to 2022 network construction cohort and the 2023 training set and then applied the trained fixed model to the 2023 test set for missing value imputation, which ensured complete isolation of data between the training and test sets.

As readmission within 30 days of discharge typically exhibits a strong clinical correlation with the initial hospitalization, it is considered to reflect continuous disease progression following the primary admission. Therefore, for patients with multiple hospitalization records, this study referred to the processing methods of previous health services research and merged the data using a 30-day time window rule based on unique inpatient numbers and admission and discharge dates [33,34]. The specific record integration rule was as follows: if the time interval between a patient’s previous discharge and subsequent admission was ≤30 days, they were merged into a single hospitalization event; if the time interval was greater than 30 days, they were treated as independent inpatient cases. For cases with multiple consecutive hospitalizations where the intervals between all adjacent admissions met the ≤30-day condition, this study adopted a continuous sequential merging strategy, unifying them into one complete medical event. When merging specific variables, the following principles were adhered to: for features such as gender, age, insurance type, admission year, and primary diagnosis, data from the first record within the merged period were extracted; the admission route was determined using a priority rule (ie, prioritizing emergency admissions, followed by outpatient, and finally other routes); regarding the planned readmission within 31 days indicator, if any record within the merged group was marked as “yes,” the entire merged event was classified as “yes”; and the discharge disposition was based on the status at the time of the final discharge. For secondary diagnostic codes, the system aggregated all records within the group and performed deduplication to construct a complete, comprehensive diagnostic set for the patient. Furthermore, the total hospitalization costs and LOS were calculated by summing all records within the merged group. In this merging process, a total of 396 patients accounted for 1096 repeat hospitalization records; through the aforementioned rules, 518 records were retained individually, and the remaining records were merged into 132, ultimately forming 650 hospitalization event records.

**Figure 1.**
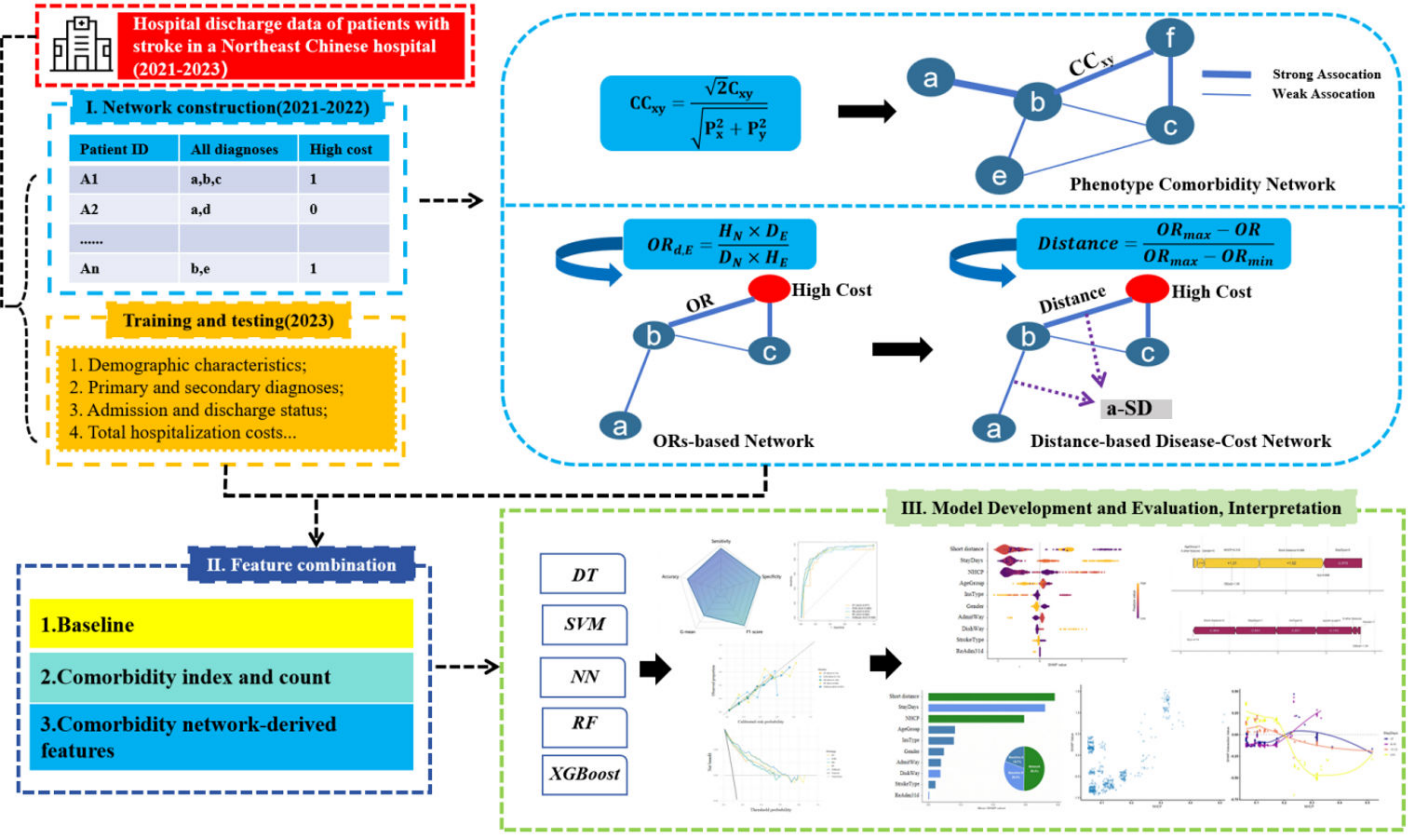
Study design and workflow for identifying inpatients with stroke who had high hospitalization costs. This retrospective study used hospital discharge data of inpatients with stroke from a tertiary hospital in Northeast China (2021‐2023). The workflow consists of three phases: (1) Network construction: data from 2021 to 2022 were used to construct comorbidity networks and extract network features. (2) Feature combination: clinical baselines, conventional comorbidity indices, and the derived network features were integrated for the 2023 cohort. (3) Model development and evaluation: The 2023 dataset was partitioned to train and test 5 machine learning models. Model performances were evaluated, and the Shapley Additive Explanations framework was applied for feature interpretability. Note: a, b, c... represent patient diseases. In the high cost column, “1” indicates a high-cost patient, and “0” indicates otherwise. DT: Decision Tree; NN: Neural Network; OR: odds ratio; RF: Random Forest; SVM: Support Vector Machine; XGBoost: Extreme Gradient Boosting.

Following the aforementioned screening and processing, this study ultimately included 10,556 patients with stroke, of which 6618 were from 2021 to 2022 and 3938 were from 2023. To prevent data leakage, this study adopted a time span–based data partitioning strategy: data from 2021 to 2022 were used to construct the comorbidity network and extract topological features (the definition of high-cost nodes within the network was also determined entirely based on data from this period); meanwhile, data from 2023 were independently divided into training and testing sets. It should be specifically noted that the network features extracted on this basis are essentially supervised risk representations based on historical data, but no information related to the 2023 data was used during their derivation process. In addition, to eliminate measurement bias caused by macroeconomic fluctuations and inflation, this study uniformly adjusted the total hospitalization costs for 2021 and 2022 to the 2023 value level based on the Consumer Price Index. The complete research flowchart is shown in Figure 1.

### Ethical Considerations

This study was approved by the Scientific Research Ethics Committee of Hangzhou Normal University (approval 2025‐1026). All procedures strictly complied with the Declaration of Helsinki and relevant ethical guidelines. Data were collected retrospectively through medical record reviews. To ensure patient privacy, all data were deidentified prior to analysis.

### Network Construction and Feature Extraction

Prior to network construction, this study established a minimum prevalence threshold, based exclusively on the 2021 to 2022 network construction cohort. Specifically, only diseases with ≥5 affected individuals within this cohort were included as network nodes, thereby ensuring the stability of the constructed network and the statistical representativeness of the extracted features. Additionally, this study incorporated the patients’ primary and secondary diagnosis codes in the early stage after admission into the network construction process. Considering the extremely high prevalence of the primary diagnosis within the study cohort, to avoid its potential computational bias on the assessment of disease associations, this study performed corresponding statistical corrections in the subsequent calculation formulas for feature indicators.

### Phenotypic Comorbidity Network

In this network, nodes represent the included disease categories, and edges represent the pairwise correlations between them. Previous studies have shown that the choice of association measure can significantly affect network topology and research findings [26]. Therefore, this study used a co-occurrence correlation as the indicator to quantify the strength of association between disease pairs [35]. Compared with traditional measurement methods, the core advantage of this calculation formula lies in its denominator, which uses the arithmetic square root of the sum of the squared occurrence frequencies of each disease. This characteristic makes the indicator more robust when dealing with extremely unbalanced occurrence frequencies of 2 diseases; even if a certain disease is relatively rare, as long as it has a very high co-occurrence ratio with another high-frequency disease, this formula can still objectively assign it a reasonable association weight. To filter out noisy edges caused by coincidental co-occurrences, this study set a minimum edge weight screening mechanism, retaining only associations with an edge weight >0.01. The final constructed PCN contained 139 valid nodes and 2404 valid edges, with a network density of 0.251. The specific calculation formula is defined as follows:


$$CC_{xy}=\frac{\sqrt{2C_{xy}}}{\sqrt{P_{x}^{2}+P_{y}^{2}}}$$

**Figure 2.**
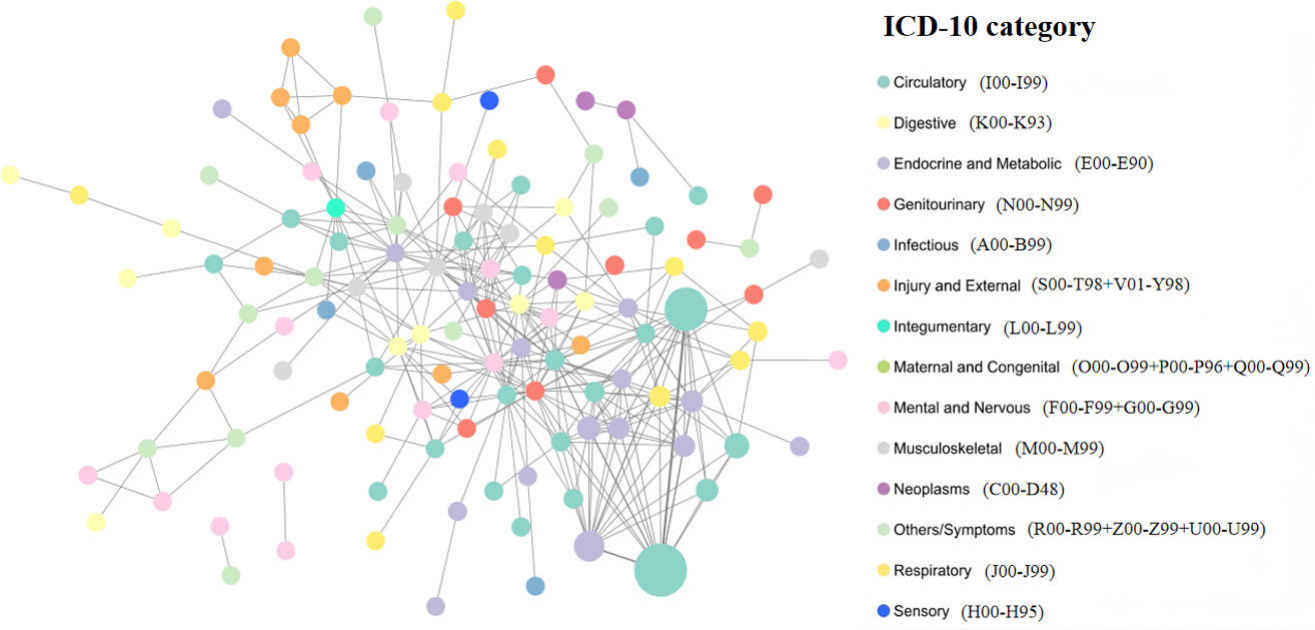
Schematic representation of the phenotype comorbidity network. Node colors denote different *International Classification of Diseases, Tenth Revision* (*ICD-10*) categories. The thickness of the edges indicates the strength of the correlation, and node size represents disease prevalence.

Here, *C_xy_* refers to the co-occurrence frequency of diseases *x* and *y* within each patient, while *P_x_* and *P_y_* represent the prevalence rates of diseases *x* and *y*, respectively. Figure 2 illustrates the generated PCN; to optimize visualization, the network displays only edges with weights ≥0.1.

### Distance-Based Disease-Cost Network

Before constructing the distance-based disease-cost network (DDCN), this study first built an initial network, specifically using the OR to quantify the strength of association between nodes. Unlike the aforementioned network, this network introduced a specific “high-cost” node in addition to the conventional disease nodes, aiming to further explore the association patterns between specific diseases and high medical expenditures. The calculation formula is defined as follows:


$$OR_{d,E}=\frac{H_{N}\times D_{E}}{D_{N}\times H_{E}}$$

In this framework, *E* represents the specific high-cost outcome. *D_E_* and *D_N_* represent the number of high-cost and non–high-cost patients, respectively, in the patient subgroup with the specific comorbidity *d*. Accordingly, *H_E_* and *H_N_*, respectively, represent the number of patients corresponding to high-cost and non–high-cost states in the control subgroup (ie, patients without the comorbidity *d*). As disease co-occurrence relationships are symmetric, the constructed DDCN is an undirected graph. To address the issue of undefined OR values caused by zero cells in the 2×2 contingency table, this study introduced the Haldane-Anscombe correction, which adds 0.5 to all 4 cells of the contingency table to avoid division-by-zero errors and smooth extreme values generated by small samples. To ensure the high specificity of the network edges, the network only retained significant association edges with an OR >1 and a lower bound of its 95% CI >1. Subsequently, this study used the min-max normalization formula to transform the ORs into distance metrics, thereby completing the construction of the DDCN. Through this formula, all association strengths were mapped into the standardized distance space of [0, 1], effectively smoothing the extreme numerical fluctuations associated with the extremely high prevalence of the primary diagnosis. The finally constructed DDCN contained 140 valid nodes and 1713 valid edges, with a network density of 0.176.


$$Distance=\frac{{OR}_{\max}-OR}{{OR}_{\max}-{OR}_{\min}}$$

### Feature Engineering and Outcome Variables

#### Network Features

##### Normalized High-Cost Propensity

Derived from the PCN, this topological feature aims to quantify the cumulative risk associated with high medical expenditures for a specific disease and its adjacent network nodes [31,35]. Specifically, this indicator effectively captures direct and indirect risk association patterns: if a patient presents with a characteristic disease of the high-cost group, or if their disease exhibits significant topological connectivity with highly prevalent comorbidities in that population, the likelihood of the patient exhibiting a high-cost status increases significantly. The calculation formula for the normalized high-cost propensity (NHCP) of disease *d* is defined as follows:


$${NHCP}_{d}=\Omega_{d}+\sum_{c\in C}\left(\frac{CC_{dc}\;\Omega_{c}}{\sum_{c\in C}CC_{dc}}\right)$$

In this calculation formula, *Ω_d_* represents the proportion of high-cost patients among cases with disease *d*, *CC_dc_* represents the edge weight between disease *d* and its co-occurring disease c within the PCN, and C constitutes the set of all adjacent nodes directly connected to node *d*. Mechanistically, *Ω_d_* quantifies the inherent and direct high-cost risk associated with disease *d*, while the second term of the formula evaluates the indirect risk brought about by the network spillover effect. By introducing a normalization mechanism through dividing by the denominator *∑_c∈C_CC_dc_* (ie, the sum of all edge weights connected to *d*), the risk metrics of different nodes are standardized to a uniform scale; this effectively offsets the influence spillover of the primary diagnosis caused by its massive number of connections, enabling the indicator to more objectively reflect the true intensity of comorbidity risks rather than being solely dictated by the absolute number of node connections. As minimum disease prevalence thresholds and edge weight filters were established prior to network construction, certain ICD codes from 2023 might not be incorporated into the network; for such marginal conditions or specific rare diseases, we assumed no additional network connectivity risk and assigned them an NHCP of 0. Ultimately, the NHCP for a patient’s diagnostic set *D* is determined as follows:


$$NHCP=\max_{d\in D}\left({NHCP}_{d}\right)$$

##### Shortest Distance

Shortest distance is a global risk indicator extracted from the DDCN. Distinct from the isolated analysis of a single disease, this indicator uses Dijkstra’s algorithm to calculate the weighted shortest path from a specific disease node to the “high-cost” node, thereby quantifying the topological reachability of a disease leading to a high-cost status via the comorbidity network. The edges fed into the algorithm incorporate both “disease-disease” and “disease-cost” associations, allowing diseases that lack direct links to the high-cost node to establish indirect connections via bridging nodes. As shown in Figure 1, although the direct association between specific disease node *a* and the high-cost node may not reach statistical significance, node *a* exhibits a strong association with node *b*, and node *b* itself is closely related to high medical expenditures. Therefore, node *a* can establish a potential indirect connection to the high-cost status through node *b* acting as a mediating bridge. For isolated nodes lacking an effective connectivity path to the specific “high-cost” node after screening, this study adopted an extreme-value penalty strategy for assignment: extracting the finite maximum shortest distance length in the current network and adding a constant penalty term (+1) to assign to such isolated nodes. Numerically, this operation maps them to the position furthest from the high-cost node, thereby objectively reflecting their relatively low association strength. Similarly, out-of-network nodes appearing in the 2023 data were also assigned this extreme distance. Ultimately, the shortest distance feature value for the patient’s comprehensive diagnostic set *D* is determined as follows:


$$Shortest\;distance=\min_{d\in D}\left({shortest\;distance}_{d}\right)$$

### Comorbidity-Related Features

In addition to the topological features extracted from the comorbidity network, this study also extracted comorbidity-related features similarly derived from patients’ diagnostic coding data to compare the impact of different feature subsets on the model’s identification efficacy in subsequent analyses. Specifically, these features include the number of comorbidities, the CCI, and the ECI.

### Baseline Features

This study extracted a total of 8 baseline features from the medical record front pages, specifically including gender, age, insurance type, stroke type, admission route, discharge disposition, LOS, and planned readmission within 31 days. Among these, the insurance type was divided into 4 categories based on the local medical insurance pooling management structure: municipal insurance (applicable to the insured population in the municipal pooling area where the medical institution is located), provincial insurance (corresponding to the insured population in the provincial pooling area), out-of-town insurance (referring to the situation where the insured location and the treatment location belong to different administrative regions), and others (covering various special forms of medical security). The stroke type was defined based on the primary diagnosis code: cases with codes I60 or I61 were classified as hemorrhagic stroke, and cases with code I63 were classified as ischemic stroke. The admission route was divided into emergency, outpatient, and others; the discharge disposition covered routine discharge upon medical advice, discharge against medical advice, and death. The planned readmission within 31 days was dichotomized as “yes” or “no” based on the discharge record in the hospital discharge data.

It is worth noting that among the 8 baseline features mentioned earlier, 5 features—gender, age, insurance type, stroke type, and admission way—can be obtained at the initial stage of patient admission, whereas discharge disposition, LOS, and planned readmission within 31 days can only be finally determined at the hospital discharge stage, exhibiting an obvious information lag. Accordingly, in the subsequent analysis, we excluded these 3 lagging features and additionally developed a prediction model specifically for patients in the early stage of hospitalization to investigate its performance in the early identification of high-cost patients.

### Outcome Variables

Although previous literature has not reached a complete consensus on the definition of high-cost status, using the top 10th percentile as a classification benchmark has been widely recognized and adopted [36,37]. Consequently, this study defined cases ranking in the top 10% of total hospitalization costs within the study cohort as high-cost patients. Furthermore, the detailed components of inpatient costs were solely used to define the outcome variable and were strictly excluded from subsequent analyses. The selection of this threshold was mainly based on the following considerations: on the one hand, this criterion can effectively alleviate the extreme data imbalance problem associated with setting a threshold too strictly, thereby ensuring the efficacy of subsequent statistical tests and classification evaluations; on the other hand, this criterion can also prevent the dilution of the typical distribution characteristics of the high-cost group caused by a definition scope that is too broad. Furthermore, from the macro perspective of medical policy, the top 10% classification can accurately cover the high-multiplier case groups closely monitored under China’s current medical insurance payment system, highly aligning with the practical governance needs of current hospital cost control and refined medical insurance management. To further verify the robustness of the research findings, we conducted expanded testing in the sensitivity analysis using other alternative thresholds.

### Model Development and Comparison

This study used the 2023 data subset for the construction and validation of models. To ensure the consistency of the target variable distribution between the training and testing sets, this study adopted the hold-out method, performing a stratified random splitting based on the outcome variable at a ratio of 8:2. After splitting, a training set of 3151 cases (including 315 high-cost cases) and an independent testing set of 787 cases (including 78 high-cost cases) were ultimately generated. Aiming at the class imbalance problem existing between high-cost and non–high-cost cases, this study strictly applied the Synthetic Minority Over-sampling Technique (SMOTE) only within the training set before formally training the models. To rigorously prevent overestimation of model performance due to data leakage, no global oversampling was performed prior to cross-validation. Instead, the SMOTE algorithm was specifically embedded within the resampling pipeline. Specifically, during each iteration of the 5-fold cross-validation, SMOTE was dynamically applied only to the training folds, whereas the validation fold always retained the original real-world data distribution. This strategy effectively balanced the class distribution, mitigated the classification bias of machine learning algorithms toward the majority class, and ensured the objectivity of model evaluation. On the basis of the processed data, this study constructed 5 machine learning models to identify high-cost stroke inpatient cases, specifically covering Decision Tree (DT), Support Vector Machine (SVM), Neural Network (NN), Random Forest (RF), and Extreme Gradient Boosting (XGBoost). To guarantee the reproducibility of the experiment and effectively control the risk of overfitting, the model’s hyperparameter optimization process used a grid search strategy combined with 5-fold cross-validation strictly executed within the training set. This was done to objectively screen and determine the optimal parameter combination for each model. During the hyperparameter tuning phase, specific search spaces were tailored to the architecture of each model. For DT, the cost-complexity parameter was primarily adjusted. For SVM, both the Radial Basis Function kernel parameter and the regularization penalty were optimized. For NN, the optimization focused on the number of hidden nodes and the weight decay. For RF, the number of candidate variables per split and the minimum node size were fine-tuned. Finally, for XGBoost, a simultaneous optimization was performed on core parameters, including the number of boosting iterations, maximum tree depth, learning rate, and minimum child weight. To balance computational efficiency and mitigate the risk of overfitting, the grid search was restricted to predefined boundaries. Consequently, the identified parameters represent local optima within these ranges rather than exhaustive global optima. The identification performance of all final models was objectively evaluated on the single-split independent hold-out testing set, combined with 1000 Bootstrap resampling to obtain the 95% CIs for various evaluation metrics. To comprehensively measure the overall identification ability of the models, this study adopted the following multidimensional evaluation metric matrix: the area under the curve (AUC), sensitivity, specificity, accuracy, G-mean index, and *F*_1_-score [38]. Addressing the class imbalance characteristic commonly found in medical data, this study specifically introduced the G-mean and *F*_1_-score to more accurately and robustly reflect the overall balanced performance of the model in simultaneously handling the identification of both minority and majority classes. Furthermore, to objectively evaluate the potential gain of network topological features on the model’s identification performance, this study used the DeLong test to conduct a statistical examination of the AUC differences between models containing only baseline features and composite models integrating both baseline and network features.

In addition to the aforementioned evaluation metrics, this study further introduced calibration curves and the Brier score, aiming to evaluate the consistency between the high-cost identification probabilities output by the model and the actual observed true proportions. Finally, this study used decision curve analysis (DCA) to evaluate the net clinical benefit that the model can generate across a wide range of threshold probabilities, thereby verifying its practical application value in medical management and decision-making. Furthermore, to deeply explore the incremental value of the extracted network topological features, this study also implemented an ablation comparative analysis to systematically compare the objective differences in identification performance between “models containing only baseline clinical features” and “models further integrating traditional comorbidity features or network features.”

### Model Interpretability

To deeply analyze the key associated features behind the high medical expenditures of inpatients with stroke, this study introduced the SHAP technique to provide post-hoc interpretability analysis for the optimal-performing machine learning model. Given that the XGBoost model ultimately used for feature attribution is a tree-based ensemble algorithm, this study specifically selected the TreeExplainer optimized for such algorithms in the analysis to accurately calculate the local and global explanations of features. On the basis of co-operative game theory, the SHAP method can theoretically and fairly distribute the model’s identification results among all input variables. Under this framework, the global importance of a feature is quantified by the average level of its absolute SHAP values across the entire study cohort. Specifically, a positive SHAP value indicates that the feature is associated with an increased probability of a case being classified as “high cost,” while a negative SHAP value denotes a decrease in this classification probability.

### Statistical Analysis

This study used R (version 4.4.2; R Core Team) for related statistical analyses and plotting. In the comparative analysis of features between groups, the distribution characteristics of categorical variables were expressed as frequencies and percentages, and the differences between the high-cost and non–high-cost groups were compared using the chi-square test; continuous variables underwent a normality test prior to analysis, where data conforming to a normal distribution were presented as mean (SD), and intergroup comparisons were conducted using the independent samples *t* test; continuous variables not conforming to a normal distribution were represented by the median and IQR, and intergroup comparisons adopted the Mann-Whitney *U* test. The visualization of the PCN was completed in Cytoscape (Cytoscape Consortium).

## Results

### Patient Characteristics

**Figure 3.**
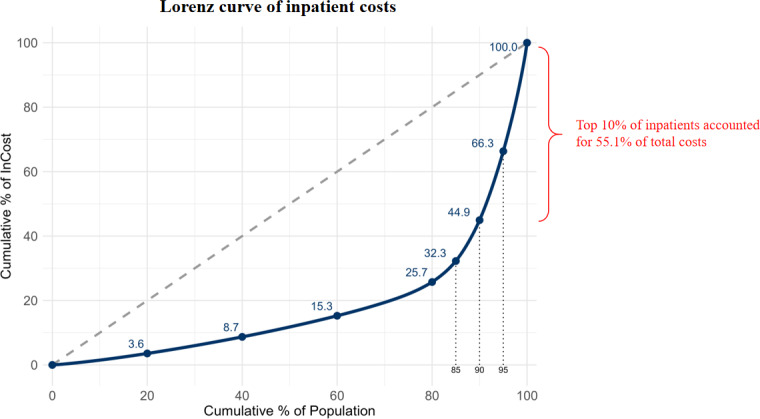
Lorenz curve of inpatient costs in 2023 revealing that the top 10% of inpatients accounted for 55.1% of total costs.

According to the previously defined high-cost threshold criteria (the top 10%, equating to ≥94,696.79 RMB), of the 3938 inpatient with stroke incorporated into the 2023 analysis, 393 cases were assigned to the high-cost cohort, and the remaining 3545 cases comprised the non–high-cost cohort. Figure 3 displays the distribution of inpatient costs among patients with stroke, with the total health care expenditures of the high-cost group making up 55.1% of the overarching cohort’s total costs.

The results of the intergroup analysis presented in Table 1 reveal that the high-cost and non–high-cost groups exhibit statistically significant differences across various features. Specifically, regarding demographics, the proportion of female patients in the high-cost group was significantly higher than that in the non–high-cost group; in terms of age distribution, the proportions of patients aged 51 to 60 years and 61 to 70 years in the high-cost group were slightly higher than those in the non–high-cost group, whereas the proportion of the population aged over 71 years in the high-cost group was distinctly lower than in the non–high-cost group. In terms of clinical features, stroke types exhibited substantial intergroup differences. Ischemic stroke predominated in the overall population, while hemorrhagic stroke accounted for 57% of the high-cost group, a proportion significantly higher than the 14.9% observed in the non–high-cost group. Furthermore, the LOS showed a strong positive correlation with high-cost status. The proportion of patients with provincial insurance among high-cost cases was markedly higher than in the non–high-cost group. Regarding network features, the intergroup comparison revealed that the NHCP value was significantly elevated in the high-cost group; conversely, the short distance value in this group was significantly reduced. As for comorbidity features, the results indicated that the high-cost patient group had a higher proportion of lower CCI scores and fewer comorbidities compared to the non–high-cost group.

**Table 1.** Characteristics of patients with stroke with high versus non–high hospital costs.

Type and characteristic	Non–high-cost group (n=3545), n (%)	High-cost group (n=393), n (%)	*P* value
Baseline			
Gender			<.001
Female	1246 (35.1)	183 (46.6)	
Male	2299 (64.9)	210 (53.4)	
Age			<.001
≤50	452 (12.8)	66 (16.8)	
51‐60	814 (23.0)	110 (28.0)	
61‐70	1290 (36.4)	148 (37.7)	
≥71	989 (27.9)	69 (17.6)	
Length of stay			<.001
≤7	927 (26.1)	64 (16.3)	
8‐10	1622 (45.8)	64 (16.3)	
11‐13	607 (17.1)	77 (19.6)	
≥14	389 (11.0)	188 (47.8)	
Stroke type			<.001
Hemorrhagic	527 (14.9)	224 (57.0)	
Ischemic	3018 (85.1)	169 (43.0)	
Insurance type			<.001
Municipal	3032 (85.5)	270 (68.7)	
Provincial	248 (7.0)	101 (25.7)	
Nonlocal	113 (3.2)	10 (2.5)	
Other	152 (4.3)	12 (3.1)	
Admission way			<.001
Outpatient	1403 (39.6)	113 (28.8)	
Emergency	2097 (59.2)	278 (70.7)	
Other	45 (1.3)	2 (0.5)	
Discharge way			<.001
Routine	3269 (92.2)	337 (85.8)	
AMA^a^	217 (6.1)	30 (7.6)	
Death	59 (1.7)	26 (6.6)	
Planned 31-day readmission			.81
No	3461 (97.6)	385 (98.0)	
Yes	84 (2.4)	8 (2.0)	
Comorbidity features			
CCI^b^			<.001
0	2072 (58.4)	291 (74.0)	
1	1211 (34.2)	86 (21.9)	
≥2	262 (7.4)	16 (4.1)	
ECI^c^			0.24
≤0	2916 (82.3)	310 (78.9)	
2‐5	414 (11.7)	56 (14.2)	
≥6	215 (6.1)	27 (6.9)	
Number of comorbidities			<.001
0‐1	1628 (45.9)	289 (73.5)	
2‐3	1423 (40.1)	59 (15.0)	
≥4	494 (13.9)	45 (11.5)	
Network features			
NHCP^d^, median (IQR)	0.07 (0.07-0.13)	0.27 (0.06-0.32)	<.001
Shortest distance, median (IQR)	2.00 (1.97-2.00)	1.00 (0.99-2.89)	<.001

a AMA: against medical advice.

b CCI: Charlson Comorbidity Index.

c ECI: Elixhauser Comorbidity Index.

d NHCP: normalized high-cost propensity.

### Model Development and Performance Comparison

Table 2 presents the identification performance of the 5 machine learning models after incorporating the 2 network features. Overall, after incorporating the network-derived features, the identification performance of all models achieved a certain degree of improvement. Among them, XGBoost exhibited the largest AUC improvement, increasing from 0.814 to 0.899; moreover, the remaining evaluation metrics of this model also demonstrated the optimal performance among the 5 models.

**Table 2.** Performance comparison of 5 machine learning models based on baseline and network-augmented feature sets. The data in parentheses are 95% CI.

Model	Feature group	
	Baseline	Baseline*+*network
DT^a^		
AUC^b^	0.824 (0.771‐0.870)	0.878 (0.835‐0.916)
Sensitivity	0.766 (0.654‐0.926)	0.893 (0.815‐0.963)
Specificity	0.803 (0.631‐0.868)	0.767 (0.675‐0.816)
Accuracy	0.799 (0.656‐0.856)	0.779 (0.703‐0.822)
G-mean	0.780 (0.731‐0.830)	0.827 (0.786‐0.863)
*F*_1_-score	0.439 (0.315‐0.532)	0.445 (0.366‐0.520)
SVM^c^		
AUC	0.829 (0.780‐0.876)	0.861 (0.809‐0.909)
Sensitivity	0.826 (0.742‐0.907)	0.810 (0.709‐0.921)
Specificity	0.792 (0.753‐0.826)	0.829 (0.701‐0.882)
Accuracy	0.795 (0.759‐0.827)	0.827 (0.718‐0.873)
G-mean	0.808 (0.764‐0.850)	0.818 (0.773‐0.863)
*F*_1_-score	0.443 (0.369‐0.513)	0.483 (0.372‐0.567)
NN^d^		
AUC	0.827 (0.774‐0.870)	0.876 (0.832‐0.916)
Sensitivity	0.804 (0.687‐0.927)	0.843 (0.744‐0.950)
Specificity	0.773 (0.611‐0.857)	0.818 (0.683‐0.879)
Accuracy	0.776 (0.642‐0.848)	0.820 (0.708‐0.873)
G-mean	0.787 (0.738‐0.831)	0.829 (0.783‐0.870)
*F*_1_-score	0.419 (0.324‐0.508)	0.486 (0.378‐0.577)
RF^e^		
AUC	0.823 (0.768‐0.869)	0.891 (0.848‐0.929)
Sensitivity	0.822 (0.671‐0.940)	0.824 (0.732‐0.912)
Specificity	0.747 (0.608‐0.882)	0.858 (0.774‐0.898)
Accuracy	0.755 (0.639‐0.864)	0.855 (0.787‐0.889)
G-mean	0.782 (0.734‐0.824)	0.840 (0.796‐0.886)
*F*_1_-score	0.402 (0.312‐0.500)	0.530 (0.425‐0.606)
XGBoost^f^		
AUC	0.814 (0.758‐0.863)	0.899 (0.857‐0.936)
Sensitivity	0.784 (0.630‐0.905)	0.826 (0.733‐0.949)
Specificity	0.770 (0.681‐0.881)	0.865 (0.698‐0.908)
Accuracy	0.771 (0.696‐0.864)	0.861 (0.723‐0.900)
G-mean	0.774 (0.725‐0.818)	0.844 (0.798‐0.891)
*F*_1_-score	0.412 (0.319‐0.522)	0.546 (0.394‐0.635)

a DT: Decision Tree.

b AUC: area under the curve.

c SVM: Support Vector Machine.

d NN: Neural Network.

e RF: random forest.

f XGBoost: Extreme Gradient Boosting.

**Figure 4.**
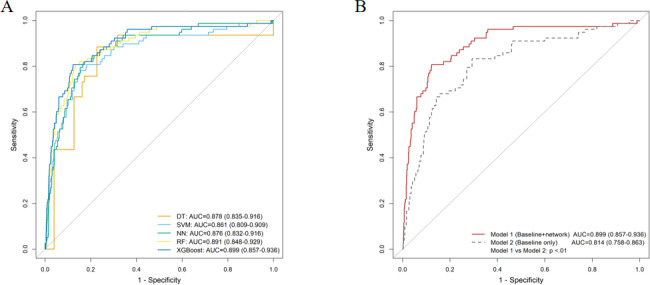
Receiver operating characteristic (ROC) curves of different machine learning models for identifying high-cost patients and comparison of feature gains. AUC: area under the curve; DT: Decision Tree; NN: Neural Network; OR: odds ratio; RF: Random Forest; SVM: Support Vector Machine; XGBoost: Extreme Gradient Boosting.

Figure 4 displays the receiver operating characteristic curves of the 5 machine learning models combined with network features; the DeLong test results in Figure 4B further confirmed that the difference in AUC gain between the “composite model fusing network features” and the “initial model containing only baseline features” is statistically significant. Specifically, the difference in AUC between the 2 models was 0.085 (95% CI 0.052‐0.123), with an exact *P* value of 1.42×10⁻⁶ according to the DeLong test.

**Figure 5.**
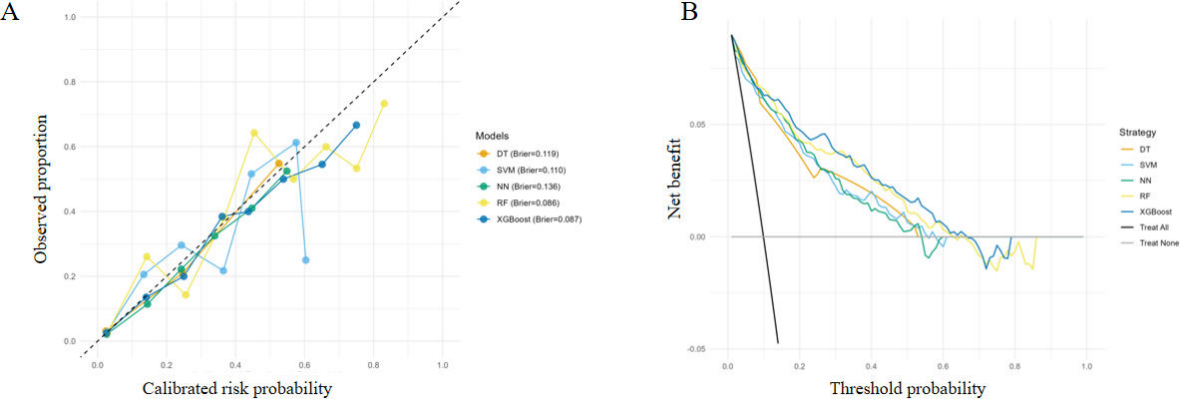
Evaluation of calibration and clinical utility of various machine learning models. AUC: area under the curve; DT: Decision Tree; NN: Neural Network; OR: odds ratio; RF: Random Forest; SVM: Support Vector Machine; XGBoost: Extreme Gradient Boosting.

The calibration curve results in Figure 5A show that the curve trajectory of the XGBoost model is closest to the ideal reference line, indicating a high degree of consistency between the high-cost identification probabilities output by the model and the actual observed true proportions. Simultaneously, XGBoost achieved a Brier score of 0.087, belonging to the low-error tier alongside RF. Its overall calibration performance was significantly better than that of the SVM, DT, and NN, further confirming the robust reliability of its output identification probability estimates. In the DCA shown in Figure 5B, XGBoost similarly highlighted significant practical application advantages. Specifically, within the broad threshold probability interval of clinical and management significance, the net benefit curve of XGBoost consistently remained at the highest level; this means that compared to other machine learning models participating in the evaluation, using this model to guide medical resource allocation can yield a more substantial comprehensive net benefit. Given the outstanding comprehensive identification performance described earlier, this study ultimately selected XGBoost as the core model for subsequent feature attribution and mechanism analysis. The optimal hyperparameter combinations and their specific configurations for each model are detailed in Table A1 in the Multimedia Appendix 1.

### Ablation Analysis

To objectively evaluate the specific contributions of traditional comorbidity features and single network topological features to the model’s identification performance, this study further implemented an ablation analysis relying on the XGBoost model. The outcomes are presented in Table 3: in terms of traditional comorbidity features, regardless of the introduction of CCI, ECI, or a basic comorbidity count, the model’s identification performance demonstrated a certain degree of enhancement; however, its overall performance still fell markedly short of the model integrating network features. In contrast, the incorporation of CCI led to a certain degree of performance degradation. Furthermore, the 2 network topological features also had different focuses regarding their gains in identification performance. Specifically, the “baseline+SD” model demonstrated better discrimination in the AUC metric; in contrast, although the “baseline+NHCP” model had a slightly lower AUC value, it performed better on the *F*_1_-score. Finally, by rigorously excluding lagging variables that could only be determined after discharge and retaining only features available in the early stage of hospitalization, we independently developed a corresponding early identification model. The results showed that, although the model achieved an acceptable AUC, its *F*_1_-score was markedly low, suggesting a potential imbalance in model performance when based solely on the currently available features.

**Table 3.** Performance evaluation of models based on single network features, traditional comorbidity features, and preadmission features. The data in parentheses are 95% CI.

Feature group	Performance					
	AUC^a^	Sensitivity	Specificity	Accuracy	G-mean	*F*_1_-score
Baseline+CCI^b^	0.807 (0.747‐0.863)	0.746 (0.608‐0.867)	0.801 (0.679‐0.905)	0.796 (0.695‐0.879)	0.771 (0.714‐0.819)	0.423 (0.329‐0.532)
Baseline+ECI^c^	0.827 (0.773‐0.874)	0.812 (0.714‐0.899)	0.774 (0.687‐0.829)	0.777 (0.702‐0.823)	0.792 (0.742‐0.836)	0.420 (0.338‐0.491)
Baseline+comorbidity count	0.852 (0.808‐0.891)	0.804 (0.690‐0.926)	0.785 (0.670‐0.864)	0.787 (0.693‐0.853)	0.793 (0.748‐0.839)	0.432 (0.336‐0.519)
Baseline+NHCP^d^	0.888 (0.845‐0.925)	0.832 (0.743‐0.910)	0.843 (0.802‐0.883)	0.842 (0.804‐0.878)	0.837 (0.793‐0.880)	0.511 (0.435‐0.591)
Baseline+shortest distance	0.894 (0.853‐0.929)	0.876 (0.756‐0.972)	0.797 (0.694‐0.893)	0.804 (0.717‐0.883)	0.833 (0.793‐0.874)	0.477 (0.372‐0.592)
Baseline-A^e^+network	0.848 (0.803‐0.890)	0.869 (0.759‐0.942)	0.718 (0.660‐0.808)	0.733 (0.681‐0.808)	0.789 (0.750‐0.829)	0.393 (0.321‐0.468)

a AUC: area under the curve.

b CCI: Charlson Comorbidity Index.

c ECI: Elixhauser Comorbidity Index.

d NHCP: normalized high-cost propensity.

e Baseline features available at admission.

Previous intergroup analysis of baseline features showed a statistically significant difference in stroke type between the 2 groups, indicating a high degree of clinical relevance between this feature and high-cost status. Given that all diagnostic codes, including the principal diagnosis, were incorporated in the construction of the comorbidity network, the resulting network topological features to some extent captured information inherent to stroke subtypes. To deeply explore the potential multicollinearity and information overlap effects among features, this study additionally constructed a model that excluded the stroke subtype and retained only the network features. The results showed that after excluding this subtype feature, the model’s identification performance was basically unaffected (relevant evaluation data are detailed in Table A2 in the Multimedia Appendix 1). In other words, the value of network features lies more in complementing existing clinical information than in providing discriminative ability that is entirely independent of stroke subtypes. Therefore, the incremental value of network features should be interpreted as a marginal improvement beyond established clinical variables, rather than as a complete replacement for conventional features. Table A2 in Multimedia Appendix 1 also summarizes the performance comparison of models fusing all feature combinations. The comprehensive analytical results corroborated that the feature combination of “baseline characteristics+dual network features” delivered the most optimal overall identification capabilities across all evaluation metrics. Therefore, this study ultimately selected this feature subset as the standard input for the subsequent SHAP feature attribution analysis.

Meanwhile, to verify the applicability and robustness of the aforementioned identification framework in specific clinical subgroups, this study further evaluated the model’s performance within the hemorrhagic and ischemic stroke subgroups in the independent testing set, based on this optimal feature combination. The stratified analysis results (Table 4) showed that although the AUC value of each model within a single subgroup slightly decreased by approximately 0.03 to 0.04 compared to the overall cohort, they still demonstrated stable identification performance across different clinical subgroups.

**Table 4.** Subgroup analysis of machine learning model identification performance across different stroke types.

Model and subgroup	AUC^a^ (95% CI)
DT^b^	
Hemorrhagic	0.828 (0.769‐0.877)
Ischemic	0.830 (0.770‐0.888)
SVM^c^	
Hemorrhagic	0.780 (0.690‐0.860)
Ischemic	0.821 (0.740‐0.895)
NN^d^	
Hemorrhagic	0.790 (0.707‐0.864)
Ischemic	0.842 (0.767‐0.905)
RF^e^	
Hemorrhagic	0.849 (0.775‐0.913)
Ischemic	0.848 (0.776‐0.907)
XGBoost^f^	
Hemorrhagic	0.868 (0.798‐0.927)
Ischemic	0.856 (0.783‐0.915)

a AUC: area under the curve.

b DT: Decision Tree.

c SVM: Support Vector Machine.

d NN: Neural Network.

e RF: Random Forest.

f XGBoost: Extreme Gradient Boosting.

### Model Interpretability

**Figure 6.**
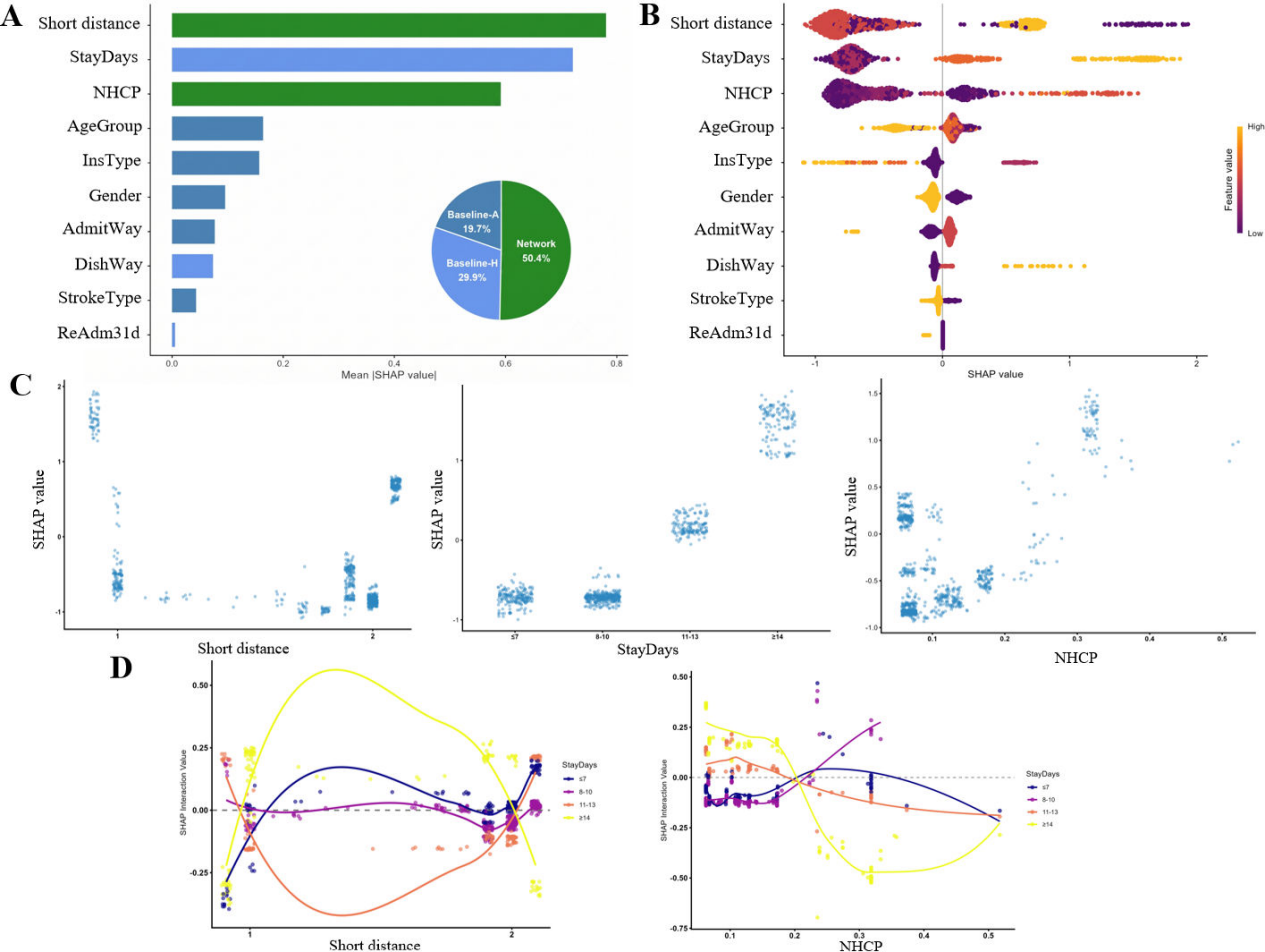
Global interpretation of the Extreme Gradient Boosting model using Shapley Additive Explanations (SHAP) analysis. (A) SHAP feature importance bar chart, Baseline-A: Baseline features available at admission; Baseline-H: Baseline features available at hospital discharge. (B) SHAP summary plot (beeswarm) showing the distribution of feature impacts. Each dot represents a sample; color indicates feature value (yellow=high and purple=low). Taking StayDays as an example, yellow dots (longer stays) are predominantly distributed on the positive SHAP side, indicating that prolonged hospitalization increases the likelihood of high-cost classification, while purple dots (shorter stays) cluster on the negative side, suggesting an inhibitory effect on high-cost generation. (C) SHAP dependence plots for top features (SD, StayDays, and normalized high-cost propensity [NHCP]), illustrating the marginal relationship between the feature value and its impact on the model output. (D) SHAP interaction plots illustrating the interaction effects of SD and NHCP with length of stay. Variable name mapping: StayDays=Length of Stay; InsType=Insurance Type; AgeGroup=Age; AdmitWay=Admission Way; DischWay=Discharge Way; ReAdm31d=Planned 31-day Readmission.

To enhance the transparency and interpretability of the model in the context of clinical decision-making, this study introduced the SHAP method to objectively quantify the marginal contribution of each input variable to the final identification result. This method covers 2 dimensions: global interpretability at the feature level and local interpretability at the individual level. In terms of global interpretability, the bar chart in Figure 6A is sorted in descending order based on the mean absolute SHAP values of the features, intuitively illustrating the relative importance of different variables in the identification of high-cost patients. The analysis results showed that the top 5 core associated features contributing to the model’s performance were, in order, short distance, LOS, NHCP, age, and insurance type. As shown in the pie chart in Figure 6A, the cumulative contribution of network features accounts for 50.4% of the model’s total output. Among the baseline features, baseline features available at admission account for 19.7%, while baseline features available at hospital discharge reach 29.9%. The SHAP beeswarm plot in Figure 6B intuitively presents the distribution trend of SHAP values across various features for the entire sample. Figure 6C displays the SHAP dependence plots for the top 3 features, showing how these variables regulate the model’s output within different value ranges. Specifically, a lower value of shortest distance, along with higher values of LOS and NHCP, is significantly associated with a high probability of cases being identified as a high-cost status. The SHAP interaction analysis in Figure 6D reveals that the interaction effects between network features and LOS exhibit complex nonlinear synergistic associations. Specifically, patients with a LOS ≥14 days show a significant inverted U-shaped trend, with the interaction value reaching a positive peak when shortest distance is at a moderate level, significantly elevating the high-cost risk; the 11‐ to 13-day group displays a U-shape with mostly negative interactions; whereas the ≤10-day group has a flat interaction effect, fluctuating around the baseline. On the other hand, when examining the interaction between NHCP and LOS: for patients with an LOS of 8 to 10 days, the interaction effect shows an upward trend, turning from a negative value to a positive value as NHCP increases. Conversely, for the subgroup with an LOS ≥14 days, the interaction effect presents a downward trend, turning negative with the increase of NHCP. In contrast, for the subgroups with an LOS ≤7 days and 11 to 13 days, the interaction values remain relatively stable across different levels of NHCP.

**Figure 7.**
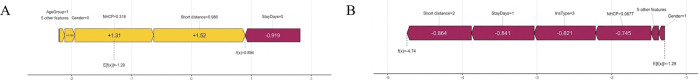
Local explanations for individual predictions using Shapley Additive Explanations force plots. The plots illustrate how specific feature values contribute to pushing the model’s output from the base value (E[f(x)]) toward the final prediction (f(x)). Yellow bars indicate features that increase the likelihood of being a high-cost patient (positive contribution), while purple bars indicate features that decrease it (negative contribution). (A) A representative high-cost patient sample (f(x)=0.894). (B) A representative non–high-cost patient sample (f(x)=−4.740). Variable name mapping: StayDays=Length of Stay; InsType=Insurance Type; AgeGroup=Age. NHCP: normalized high-cost propensity.

At the local explanation level, we generated SHAP force plots to elucidate the contribution of each feature to the identification result for a specific individual. Figure 7A displays a patient who was identified by the model as highly likely to be in a high-cost status. The core features making the primary positive contribution to this identification probability were shortest distance and NHCP, while the LOS played a certain mitigating role. Conversely, the patient in Figure 7B was identified as having an extremely low probability of being in a high-cost status. The dominant associated features supporting the identification of this case into the non–high-cost group were, in order, a higher shortest distance, a shorter LOS, a lower NHCP, and a specific insurance type.

### Sensitivity Analysis

As presented in Table 5, at the extreme threshold of 5%, the model’s AUC value reached its highest level; however, affected by the extremely imbalanced data, the model’s *F*_1_-score was below 0.4, meaning that the model performance was poor and the identification ability was relatively weak. As the threshold was relaxed, although the model’s AUC value experienced a slight decline, it showed a relatively stable trend within the 10% to 20% range. In addition, due to the steady increase in the number of positive samples, the class imbalance phenomenon was effectively alleviated, prompting the *F*_1_-scores of all models to show a gradually increasing trend. Among them, the threshold adjustment from 5% to 10% optimized the model performance most significantly, and the *F*_1_-scores of all models achieved substantial improvements. This indicates that the 10% threshold effectively improved the problem of impaired model performance caused by extreme class imbalance under the low threshold.

**Table 5.** Sensitivity analysis of model performance under varying thresholds. The data in parentheses are 95% CI.

Thresholds	Performance					
	AUC^a^	Sensitivity	Specificity	Accuracy	G-mean	*F*_1_-score
5%	0.939 (0.914‐0.963)	0.951 (0.881‐1.000)	0.847 (0.814‐0.880)	0.852 (0.820‐0.884)	0.897 (0.859‐0.930)	0.389 (0.296‐0.479)
10%	0.899 (0.857‐0.936)	0.826 (0.733‐0.949)	0.865 (0.698‐0.908)	0.861 (0.723‐0.900)	0.844 (0.798‐0.891)	0.546 (0.394‐0.635)
15%	0.906 (0.875‐0.933)	0.884 (0.760‐0.966)	0.806 (0.720‐0.914)	0.818 (0.748‐0.895)	0.842 (0.811‐0.872)	0.597 (0.508‐0.700)
20%	0.898 (0.869‐0.923)	0.894 (0.821‐0.950)	0.789 (0.728‐0.857)	0.810 (0.767‐0.858)	0.839 (0.812‐0.868)	0.653 (0.591‐0.712)

a AUC: area under the curve.

## Discussion

### Principal Findings

Unlike most previous models, which incorporated clinical laboratory, imaging, or genomic data [39-41], this study used hospital discharge data to develop a high-performance model for identifying patients with stroke who have high hospitalization costs by combining diagnostic network analysis with machine learning algorithms. Compared with these data sources, hospital discharge data offer several important advantages, including greater standardization, lower cost, and broad availability across hospitals at all levels, making it a practical basis for implementation in diverse health care settings. Although this data source has inherent limitations in dimensionality, incorporating topological features derived from the comorbidity network substantially improved the performance of all models, consistent with previous studies [30,31]. Moreover, compared with other models for identifying high-need, high-cost patients that did not include network features, our model showed superior performance [17,42-44].

Our results indicate that, although both are derived from diagnostic data, network features from the comorbidity network contributed more to model performance than conventional comorbidity measures such as CCI, ECI, and comorbidity count. By quantifying the pathway from a specific disease node to high-cost outcomes and integrating the disease’s intrinsic risk with the combined effects of its local neighborhood, these features captured latent information that is difficult to detect using traditional statistical methods, thereby substantially improving the model’s ability to identify high-cost patients. Additionally, subgroup analyses across different stroke subtypes further verify the stability and reliability of the comorbidity network features extracted in this study. The model maintained satisfactory predictive performance under the macroscopic classification of hemorrhagic and ischemic stroke, demonstrating good applicability across major clinical subgroups. Nevertheless, this classification is relatively coarse and ignores substantial clinical heterogeneity among different subtypes within hemorrhagic stroke. We therefore conducted a further refined subgroup analysis focusing on subarachnoid hemorrhage (I60) and intracerebral hemorrhage (I61), as presented in Table A3 in Multimedia Appendix 1. The results showed that the model performed stably in the I61 subgroup but yielded unsatisfactory outcomes in the I60 subgroup. This discrepancy can be explained by the sample characteristics of the I60 population. This subgroup had a small sample size and a high degree of internal homogeneity. Among the total of 247 patients with I60, 170 were categorized as high-cost cases, indicating highly consistent hospitalization costs in this subtype. Limited information from routine hospital discharge data failed to capture subtle individual differences, which impaired the model’s discriminative ability. In future research, we will expand the sample size and incorporate more clinical indicators, treatment-related information, and other variables to develop refined models for individual stroke subtypes.

In the global feature importance ranking, LOS was the second most important feature, after shortest distance. Previous studies have likewise highlighted the critical role of LOS in identifying high-cost pediatric inpatients [42]. SHAP interaction analysis further showed that the association between LOS and comorbidity network features was strongly time dependent, suggesting that the trajectory of medical resource use in stroke may evolve dynamically over the course of the disease. In the subgroup with an LOS of 8 to 10 days, higher NHCP showed a clear positive synergistic interaction, which may reflect the clinical profile of complex cases requiring highly resource-intensive interventions during the acute phase. However, when LOS was ≥14 days, high NHCP instead showed a strong negative interaction; at the same time, this long-stay subgroup exhibited a positive interaction peak at moderate levels of shortest distance. This time-varying pattern may suggest a law of diminishing marginal costs: as hospitalization becomes substantially prolonged, the main drivers of high expenditure may shift from fluctuations in acute disease severity to the cumulative baseline costs of ongoing care. Meanwhile, the 11 to 13 days subgroup showed a U-shaped pattern, with negative interactions across most of the value range, which may indicate a transitional stage in cost generation as patients move from the acute phase to the longer-term recovery phase.

Age and insurance type were also important determinants of the model’s identification performance. In particular, the effect of age on high hospitalization costs showed a nonlinear pattern, with older age exerting a negative effect on the identification of high-cost patients. One possible explanation is that because comorbid degenerative conditions increase surgical risk [45], older patients are more likely to receive conservative treatment and, compared with younger patients, are less likely to undergo high-cost interventional procedures, such as mechanical thrombectomy [46]. This pattern of clinical management is consistent with the baseline finding that patients in the high-cost group were, on average, younger. In addition, this study found a relatively high proportion of patients with a low comorbidity burden in the high-cost group. As patients in this group were generally younger, the prevalence of chronic underlying diseases would be expected to be lower, which may partly explain the clustering of relatively mild comorbidity in this group. However, given the inherent limitations of retrospective observational studies, future research should further examine the causal relationships among age, comorbidity, and high hospitalization costs in patients with stroke through prospective study designs. Notably, the performance of CCI, comorbidity count, and ECI in between-group comparisons was not entirely consistent, which may be attributable to differences in disease composition and weighting structure among these comorbidity measures. Comorbidity count reflects only the number of coexisting conditions and does not incorporate disease-specific weights. In contrast, the CCI applies weights based on a relatively limited set of comorbidity categories, whereas the ECI encompasses a broader range of comorbidity conditions and is based on a different scoring framework. Regarding insurance type, patients covered by provincial medical insurance generally benefit from higher reimbursement rates and broader formulary coverage. As a result, they may have greater financial access to high-cost diagnostic and therapeutic services and, when treated at tertiary grade A hospitals, may be more likely to accept more comprehensive or resource-intensive interventions. This may explain the significant positive association between provincial medical insurance and high-cost status. In contrast, patients with cross-provincial medical insurance or other payment methods may be constrained by reimbursement limits or out-of-pocket affordability. In clinical decision-making, these patients may therefore be more likely to adopt more economical or conservative treatment strategies, which is reflected in the model as a lower probability of high medical expenditure. Clinical management in these groups may prioritize cost-effective care, resulting in a negative association with high-cost status. This observed pattern is consistent with the findings of Yang et al in the Chinese ischemic stroke population [47].

Sensitivity analysis showed that when the outcome threshold was set at 5%, the constructed model performed poorly, with limited practical value for management purposes. As the threshold was relaxed from 10% to 20%, the model’s AUC tended to stabilize, while the *F*_1_-score increased. However, this improvement in *F*_1_-score may mainly reflect the passive effect of a larger number of positive samples and a more balanced class distribution, rather than a true improvement in the model’s discriminative ability. In addition, a broader threshold of 15% or 20% may dilute the core characteristics of the truly high-expenditure group, thereby creating potential challenges for the precise allocation of clinical resources and the refined management of medical insurance cost control. In contrast, a 10% threshold allows greater concentration on the core high-cost group and more accurately targets the disproportionately high-expenditure cases prioritized under the current medical insurance payment system. It should be noted, however, that at this 10% prevalence level, the optimal model yields a positive predictive value of approximately 0.41. While this implies a certain false-positive fraction, these misclassified cases typically represent moderately complex patients who may still benefit from subsequent care management. Ultimately, the practical implementation of this model relies on available administrative capacity. If hospital resources are relatively sufficient, health care managers can effectively absorb the interventions for these false positives and may even appropriately increase the threshold to expand coverage, thereby minimizing the risk of overlooking other potential high-cost cases.

### Limitations and Future Directions

First, the training and test sets in this study were generated using a single stratified random split, and the model evaluation results may therefore be influenced by variability arising from random sample partitioning. Although bootstrap resampling was used to estimate CIs, it could not fully eliminate the bias inherent to a single split. Due to the constraints of the study design for temporal validation and to preserve the original temporal characteristics of the cohort, repeated data splitting was not performed. Future studies could further enhance the reliability of the results through repeated split-sample validation. Second, the data were primarily sourced from a single center, which may introduce biases related to region-specific medical practices or insurance policies. Future research should prioritize multicenter external validation to confirm the model’s robustness; furthermore, the generalizability of this network feature-based framework to other disease spectra remains to be validated. Third, given the retrospective nature of this study and its reliance on hospital discharge data, some features exhibit information lag, which limits the utility of the model for preadmission screening. Although we used features available at admission to construct an early identification model, achieving more precise screening will require incorporating other features available at admission based on this study. However, SHAP-based interpretability analysis still holds significant value in providing decision support for clinical process management and guiding personalized interventions. Additionally, a specific limitation exists regarding the “planned readmission within 31 days” indicator. Owing to our 30-day aggregation strategy, merged readmission episodes accrue higher total costs, introducing a potential circularity issue that could theoretically inflate this feature’s apparent contribution. Although our SHAP analysis revealed it had the lowest importance contribution—indicating a minimal practical impact on the model’s identification performance in this cohort—future studies should carefully account for this potential confounding effect when constructing cost-related variables. Fourth, although the combination of comorbidity network features enriches the feature space, the current absence of biomarkers may limit the granularity of disease severity representation. Future research should prioritize the development of multimodal fusion models to evaluate the incremental value of biomarkers relative to network topological features, thereby establishing a more comprehensive and multidimensional risk profile for patient expenditures. Fifth, the static network constructed in this study may not fully capture high-order dependencies or the dynamic trajectories of disease evolution. Therefore, future research should integrate advanced algorithms, such as Graph Neural Networks and temporal network analysis [48,49], to unlock new opportunities for mining deep comorbidity associations and optimizing identification performance. Sixth, certain limitations remain in our data processing. Continuous variables were neither standardized nor normalized prior to KNN imputation and model training. Although our optimal model, XGBoost, is invariant to feature scaling, the lack of normalization may have impaired the performance of distance-based algorithms, such as SVM and NN. Additionally, despite a low missing data rate (0.13%‐1.95%), applying KNN imputation without prior scaling is a methodological limitation that may introduce minor computational biases. Future studies using distance-sensitive algorithms should incorporate a comprehensive data scaling pipeline. Finally, total hospitalization costs were adjusted for economic fluctuations using the Consumer Price Index. As the general Consumer Price Index primarily tracks a standard basket of consumer goods and medical inflation often outpaces general inflation, this approach may slightly misestimate the true medical cost inflation.

### Conclusions

By integrating comorbidity network analysis with machine learning, this research used hospital discharge data to develop a high-performance framework for the identification of high-cost stroke patients. Our results confirm that, compared to stroke type within baseline features and conventional comorbidity features, network topological features are able to capture deeper information regarding disease complexity. The inclusion of network features improved model performance, with the XGBoost model demonstrating the best identification performance. Furthermore, SHAP analysis identified strong associations between high costs and relevant features while also revealing complex nonlinear interactions among these variables. These findings indicate that this framework demonstrates promising application prospects for cost-risk stratification in patients with stroke. If validated externally and prospectively in the future, this model could provide valuable decision support for exploring early identification of medical costs and stratified intervention strategies, thereby assisting in the optimization of medical resource allocation.

## Supplementary material

10.2196/93680 Multimedia Appendix 1 Supplementary data regarding the configuration and evaluation of the machine learning models, specifically containing the hyperparameter search spaces and the resulting optimal parameters for the machine learning models; a forest plot illustrating the classification performance for the models across different stroke types.

## Abbreviations

AUC: area under the curve
CCI: Charlson Comorbidity Index
DDCN: distance-based disease-cost network
DT: Decision Tree
DVA: decision curve analysis
ECI: Elixhauser Comorbidity Index
*ICD-10*: *International Classification of Diseases, Tenth Revision*
KNN: K-Nearest Neighbors
LOS: length of stay
NHCP: normalized high-cost propensity
NN: Neural Network
OR: odds ratio
PCN: phenotypic comorbidity network
RF: Random Forest
SHAP: Shapley Additive Explanations
SMOTE: Synthetic Minority Over-sampling Technique
SVM: Support Vector Machine
XGBoost: Extreme Gradient Boosting
