# Supplementary material for: Network Analysis–Driven Machine Learning Model for Identifying High-Cost Stroke Inpatients Using Hospital Discharge Data: Retrospective Study
Source: JMIR Med Inform. 2026 Aug 10;14:e93680. doi: 10.2196/93680 (PMC13456307; doi:10.2196/93680)
Supplement: Supplementary file 1 [file medinform-v14-e93680-s001.docx]

| *Model* | *Search Space* | *Optimal Parameters* |
| --- | --- | --- |
| *DT* | [cp] range: 0.005 to 0.1 (4 steps total) | cp = 0.01 |
| *SVM* | [sigma] range: 0.01 to 0.1 (3 steps total) \| [C] range: 0.5 to 5 (3 steps total) | sigma = 0.01; C = 5 |
| *NN* | [size] range: 15 to 20 (2 steps total) \| [decay] range: 0 to 1e-04 (2 steps total) | size = 20; decay = 0 |
| *RF* | [mtry] range: 2 to 4 (2 steps total) \| [splitrule] value: gini \| [min.node.size] range: 1 to 5 (2 steps total) | mtry = 2; splitrule = gini; min.node.size = 1 |
| *XGBoost* | [nrounds] range: 150 to 300 (2 steps total) \| [max_depth] range: 3 to 5 (2 steps total) \| [eta] range: 0.05 to 0.1 (2 steps total) \| [gamma] range: 0 to 0 (1 step total) \| [colsample_bytree] range: 0.8 to 0.8 (1 step total) \| [min_child_weight] range: 1 to 3 (2 steps total) \| [subsample] range: 0.8 to 0.8 (1 step total) | nrounds = 150; max_depth = 3; eta = 0.05; gamma = 0; colsample_bytree = 0.8; min_child_weight = 3; subsample = 0.8 |

Table A1. Hyperparameter search space and optimal parameters for machine learning models.

Table A2. Performance of machine learning models under different feature combinations.

| *Model* | *Feature Group* | |
| --- | --- | --- |
|  | *Baseline(Exclude Stroke type)+Network* | *Baseline+All feature group* |
| *DT* |  |  |
| AUC | 0.873 (0.832-0.912) | 0.876 (0.836-0.916) |
| Sensitivity | 0.890 (0.805-0.973) | 0.883 (0.729-0.983) |
| Specificity | 0.754 (0.664-0.814) | 0.758 (0.667-0.892) |
| Accuracy | 0.768 (0.691-0.820) | 0.771 (0.693-0.883) |
| G-mean | 0.818 (0.777-0.857) | 0.814 (0.779-0.856) |
| F1-score | 0.433 (0.347-0.512) | 0.444 (0.344-0.578) |
| *SVM* |  |  |
| AUC | 0.883 (0.840-0.922) | 0.889 (0.853-0.922) |
| Sensitivity | 0.836 (0.739-0.929) | 0.834 (0.735-0.949) |
| Specificity | 0.846 (0.772-0.905) | 0.823 (0.664-0.895) |
| Accuracy | 0.845 (0.781-0.896) | 0.824 (0.690-0.883) |
| G-mean | 0.840 (0.794-0.882) | 0.827 (0.780-0.870) |
| F1-score | 0.521 (0.413-0.624) | 0.488 (0.379-0.583) |
| *NN* |  |  |
| AUC | 0.866 (0.818-0.908) | 0.869 (0.827-0.907) |
| Sensitivity | 0.833 (0.740-0.922) | 0.821 (0.700-0.956) |
| Specificity | 0.833 (0.754-0.882) | 0.809 (0.646-0.897) |
| Accuracy | 0.833 (0.767-0.877) | 0.810 (0.675-0.886) |
| G-mean | 0.832 (0.790-0.876) | 0.812 (0.766-0.857) |
| F1-score | 0.498 (0.406-0.589) | 0.472 (0.348-0.585) |
| *RF* |  |  |
| AUC | 0.891(0.848-0.930) | 0.893 (0.852-0.928) |
| Sensitivity | 0.855 (0.763-0.932) | 0.857 (0.711-0.987) |
| Specificity | 0.840 (0.788-0.885) | 0.800 (0.647-0.925) |
| Accuracy | 0.841 (0.795-0.882) | 0.805 (0.679-0.907) |
| G-mean | 0.847 (0.805-0.887) | 0.824 (0.782-0.867) |
| F1-score | 0.517 (0.434-0.600) | 0.481 (0.352-0.621) |
| *XGBoost* |  |  |
| AUC | 0.898 (0.855-0.935) | 0.897 (0.862-0.927) |
| Sensitivity | 0.832(0.739-0.927) | 0.894 (0.779-0.973) |
| Specificity | 0.863 (0.747-0.902) | 0.785 (0.689-0.883) |
| Accuracy | 0.860 (0.765-0.897) | 0.796 (0.713-0.877) |
| G-mean | 0.847 (0.802-0.891) | 0.836 (0.799-0.871) |
| F1-score | 0.542 (0.439-0.628) | 0.468 (0.376-0.570) |

Figure A1. Forest plot of classification performance for machine learning models across stroke type.

Table A3. Subgroup Analysis of Identification Performance of Machine Learning Models by Hemorrhagic Stroke Subtype.

| **Model** | **Subgroup** | **AUC** |
| --- | --- | --- |
| DT | I60 | 0.500 (0.500-0.500) |
| SVM | I60 | 0.608 (0.459-0.783) |
| NN | I60 | 0.617 (0.455-0.800) |
| RF | I60 | 0.583 (0.444-0.750) |
| XGBoost | I60 | 0.650 (0.488-0.836) |
| DT | I61 | 0.791 (0.683-0.895) |
| SVM | I61 | 0.780 (0.597-0.922) |
| NN | I61 | 0.837 (0.724-0.930) |
| RF | I61 | 0.843 (0.703-0.947) |
| XGBoost | I61 | 0.872 (0.766-0.953) |
